# Evaluation of 16S rRNA primer sets for characterisation of microbiota in paediatric patients with autism spectrum disorder

**DOI:** 10.1038/s41598-021-86378-w

**Published:** 2021-03-24

**Authors:** L. Palkova, A. Tomova, G. Repiska, K. Babinska, B. Bokor, I. Mikula, G. Minarik, D. Ostatnikova, K. Soltys

**Affiliations:** 1 Institute of Molecular Biomedicine, Faculty of Medicine, Comenius University in Bratislava, Bratislava, Slovakia; 2 Medirex Inc., Bratislava, Slovakia; 3 Institute of Physiology, Faculty of Medicine, Comenius University in Bratislava, Bratislava, Slovakia; 4 Comenius University Science Park, Comenius University in Bratislava, Bratislava, Slovakia; 5 Hebrew University of Jerusalem, Jerusalem, Israel; 6 Department of Microbiology and Virology, Faculty of Natural Sciences, Comenius University in Bratislava, Bratislava, Slovakia

**Keywords:** Microbiome, Paediatric research, Autism spectrum disorders

## Abstract

Abstract intestinal microbiota is becoming a significant marker that reflects differences between health and disease status also in terms of gut-brain axis communication. Studies show that children with autism spectrum disorder (ASD) often have a mix of gut microbes that is distinct from the neurotypical children. Various assays are being used for microbiota investigation and were considered to be universal. However, newer studies showed that protocol for preparing DNA sequencing libraries is a key factor influencing results of microbiota investigation. The choice of DNA amplification primers seems to be the crucial for the outcome of analysis. In our study, we have tested 3 primer sets to investigate differences in outcome of sequencing analysis of microbiota in children with ASD. We found out that primers detected different portion of bacteria in samples especially at phylum level; significantly higher abundance of Bacteroides and lower Firmicutes were detected using 515f/806r compared to 27f/1492r and 27f*/1495f primers. So, the question is whether a gold standard of Firmicutes/Bacteroidetes ratio is a valuable and reliable universal marker, since two primer sets towards 16S rRNA can provide opposite information. Moreover, significantly higher relative abundance of Proteobacteria was detected using 27f/1492r. The beta diversity of sample groups differed remarkably and so the number of observed bacterial genera.

## Introduction

An advent of massively parallel sequencing (MPS) technologies has revolutionized an investigating of human microbiota^1^. MPS platforms has enabled unrevealing of the microbiota composition and bacterial identification in complex microbial samples which was not feasible before^2^. Relatively easily accessible results tempted to excitement for revealing yet unknown. However, the procedure is very complex. Obtaining the results truly reflecting the real microbiota state may require tedious optimization. All steps in DNA library preparation including sample collection and storage^3^ or DNA isolation^4^ may influence the results of analysis. Suitable protocol for DNA libraries preparation have to be chosen with regards to sequencing platform^5,6^ as well as type of specimen origin^7,8^. In this respect, primer selection seems to be the most crucial task in preparation of DNA sequencing libraries^9^ and so bacterial composition discovery. Via selection of specific amplification primers, investigating of more less any location and length of bacterial genes is enabled. Despite the fact that MPS technologies allow analysis of whole bacterial genomes, sequencing of 16S rRNA has become a gold standard in microbiota studies^10^. These genes are of suitable length and structure for phylogenetic analysis^11^. Huge advantage for 16S sequencing and subsequent bacterial identification are easily accessible and rapidly expanding databases of their sequences in specific organisms^12–14^.Various primer sets are being used for amplification of 16R rRNA genes and were considered to be universal. However, primers may differ in specificity and sensitivity. Moreover, amplification primer sets need to be chosen depending on type of biological sample as different type of microbiota can be present across various biological specimen, such as blood, saliva or stool samples^15^. New studies showed that amplification of different variable regions of 16S rRNA genes may result in different outcome and that results may be highly variable depending on primer set used. It is well-known that full length *16S rRNA* gene sequence can provide the most specific phylogenetic analysis. On the other hand, due fact that currently used MPS platforms are producing much shorter reads than the length of 16S rRNA genes, as well as due to economic aspect, shorter fragments of 16S are often chosen for analysis. These may include one or more specific variable regions. However, short amplicons may reduce specificity and sensitivity of taxonomic classification^16^. In silico study comparing several hypervariable regions of 16S rRNA genes indicates that V4-V6 region reflect full length sequences of *16S rRNA* genes the best, while V2 and V8 are the least reliable regions^17^. Sequencing analysis with results that truly mirror the real microbiota condition are of high importance as they may help to uncover connection between microbiota changes and various diseases. Recently, a lot of associations between changed microbiota conditions and health impairment such as allergic disease and asthma^18–21^, development of diabetes mellitus 1^22^, necrotizing enterocolitis^23^, inflammatory bowel disease^24^ or obesity^25,26^ have been revealed. Other studies indicate that specific microbiota composition may be associated with neurodevelopmental disorders, as ASD^27^ and schizophrenia^28^, depression^28^ or neurodegenrative disorders as Parkinson’s^29^ and Alzheimer’s disease^30^. Many studies revealed behaviour^31^ and autism spectrum disorder (ASD) particularly^32^ are linked with microbiota changes via gut-brain axis connection. According to Ho et al.^33^, there are 26 published studies regarding the microbiome composition of paediatric ASD patients. Most of them found that children with ASD have distinct gut microbiota from that in children without the condition. Unfortunately, many of these studies showed inconsistent results crosslinking only in certain taxa, including *Firmicutes* at the phylum level, *Clostridiales* clusters including *Clostridium perfringens*, *Bifdobacterium* and *Prevotella*. Also, often used marker Firmicutes/Bacteroidetes (F/B) ratio provides opposed results. The studies of Strati et al.^34^ and Williams et al.^35^ declare a significant increase in the F/B ratio in ASD subjects, some studies declare no significant changes between ASD patients and controls^36–38^. There are also studies that mention Bacteroides/Firmicutes ratio^27,34,39,40^ being increased. One of the reasons for discordances could be, different approaches applied for the ASD microbiome characterization, including 16S rRNA V3-V4 primer pair^41^, individual variable regions of 16S rRNA^42^, combined with short-read sequencing or also pyrosequencing^43^. Differences in high-throughput techniques can also implement significant changes in microbiome composition coming from the methodological approach itself.

In our study, we tested 3 different primer sets for investigating microbiota of ASD patients. For this purpose, stool was chosen as a suitable biological sample, as easily accessible in the children with ASD. The primer sets were chosen as these are standardly used for faecal microbiota investigation. Here we present the differences in microbial composition in stool samples of paediatric ASD patients that can be implemented only by using a specific primer set towards 16S rRNA. We show diametrically different low-abundance bacterial genera detected by either of the primer set. Differences in bacterial composition can be observed even within the analysis of the same 16S rRNA variable regions with alternative primer set. Furthermore, not only the quality but also the quantity of the detected bacterial genera differed between three primer sets.

## Methods

### Ethical approval

This study was approved by the Ethical committee of the Comenius University Faculty of Medicine, and the University hospital in Bratislava, Slovakia and it is consistent with the 1964 Helsinki declaration and its later amendments. Parents were aware of whole design of the study and the informed consent form was signed by both (or at least one if both are not available) parents or caregivers of the corresponding child.

The authors confirm that all methods and experimental protocols were performed in accordance with the relevant guidelines and regulations and were approved by the of the committee of the Ministry of Health of the Slovak republic.

### Experimental design

In this experiment, intestinal microbiota composition of 10 children with ASD was examined (samples A-J), while 3 different ways of sample processing were used and compared. This study was approved by the Ethical committee of the Comenius University Faculty of Medicine, and the University hospital in Bratislava, Slovakia and it is consistent with the 1964 Helsinki declaration and its later amendments. Parents were aware of whole design of the study and the informed consent form was signed by both (or at least one if both are not available) parents or caregivers of the corresponding child.

### Faecal samples

In the study, 10 boys with ASD in age 5.00 ± 0.20 (mean ± SEM), ranging from 2.8 to 9.2 years, were included. All subjects were medication–free. The diagnosis of ASD was determined by a clinical psychologist or a psychiatrist according to ICD-10 and DSM-5. The children also underwent behavioural testing by trained examiners at the Academic Research Centre for Autism, Institute of Physiology, Faculty of Medicine, Comenius University. The diagnostic tools involved: observation of a child by the Autism Diagnostic Observation Schedule- second revision (ADOS-2)^44^ and the Autism Diagnostic Interview-Revised (ADI-R)^45^, a comprehensive interview administered to parents that provides a thorough assessment of individuals with ASD. All children enrolled in the study had to meet the criteria for ASD within both autism scales.

Stool specimens were collected by parents into sterile containers, after being given a detailed explanation of the procedure and kept at 4 °C and delivered to the laboratory within 4 h. Subsamples of 200 mg of each specimen were frozen at − 80 °C until DNA extraction.

### DNA isolation

Total DNA was extracted from 200 mg of stool by QIAamp DNA Stool Mini Kit, (Qiagen, Hilden, Germany), according to the manufacturer's instructions with the final elution volume 100 μl of nuclease-free water. Isolated DNA was stored at − 80 °C until analysis.

### 16S rRNA amplification

In the study, 3 different primer sets (Table 1) were used for the amplification and further sequence analysis of *16S rRNA*. For amplification by PCR, 3–50 ng of template DNA was used. The PCR was performed in final volume 20ul, while 4ul of 5 × HOT FIREPol Blend Master Mix (Solis BioDyne, Tartu, Estonia), 0.4 ul of 10 µM both forward and reverse primers (each in final concentration 0.2 µM). PCR conditions were optimized for primer set#1 as follows: initial denaturation 95 °C/15 min, followed by 25 cycles of denaturation 95 °C/20 s, aneling 62 °C/30 s and polymerization 72 °C/2 min. After that, final polymerization was carried out at 72 °C/10 min. For primer sets 2 and 3 annealing at 60 °C for 30 s was performed. PCR products were purified using DNA Clean &Concentrator (Zymo Research, Irvine, USA).

**Table 1:** Nucleotide sequences of PCR primers.

	Primer name	Sequence	Length of PCR product (bp)	References
set#1	515f	5´-GTGCCAGCMGCCGCGGTAA-3´	292	Caporaso et al. 2011^63^
	806r	5´-TAATCTWTGGGVHCATCAGG-3´		
set#2	27f	5´-AGAGTTTGATCMTGGCTCAG-3´	1463	Lane, 1991^64^
	1492r	5´-GGTTACCTTGTTACGACTT-3´		
set#3	27f*	5´-GAGAGTTTGATCCTGGCTCAG-3´	1466	Melničáková et al. 2013^65^
	1495r	5´-CTACGGCTACCTTGTTACGA-3´		

### DNA sequencing library preparation

Short fragments (amplified by set#1 primers) were used directly for indexing PCR using Nextera XT Index Kit (Illumina, San Diego, California, USA) and Agilent SureSelect QXT Library Prep Kit (Agilent Technologies, Santa Clara, USA) and amplified according to the original protocol.

Long fragments (amplified by primer sets 2 and 3) were transposon-tagmented using Nextera XT DNA Library Preparation Kit (Illumina Inc, San Diego, California, USA) prior the indexing. For indexing, Nextera XT index kit (Illumina Inc, San Diego, California, USA) combined with master-mix from Agilent SureSelect QXT Library Prep Kit (Agilent Technologies, Santa Clara, USA) were used. Extended amplification program using 12 thermal cycles was used for 27f/1492r and 27f*/1495r sets of primer-based PCR product. In all experiments negative controls were included. Protocol evaluation using ZymoBiomics Gut Microbiome Standard (ZymoResearch, Irvine, CA, USA) was performed. DNA profile of sequencing libraries was verified using Agilent 2100 Bioanalyzer (Agilent Technologies, Santa Clara, USA) and High Sensitivity DNA Kit (Agilent Technologies, Santa Clara, USA) and quantified using Qubit 2.0 Fluorometer (Thermo Fisher Scientific, Waltham, USA). DNA libraries were analysed using Illumina MiSeq platform via 150 bp pair-end reads.

### Data analysis

The quality of sequencing reads was verified in FastQC tool and analysed in Geneious Prime software (https://www.geneious.com/prime/) (Biomatters, Ltd., Auckland, New Zealand). The paired reads were merged (set as paired reads) and 3´ ends of reads were trimmed (error probability limit: 0.03). Microbial profiles of samples were assessed by comparison with RDP database. Data were statistically analysed using two-way Anova test and visualized using GraphPad Prism 6.0 (GraphPad Software, La Jolla, CA, USA) and ClustVis 2.0 (https://biit.cs.ut.ee/clustvis/)^46^. As criteria of statistical significance *p* < 0.05 was considered.

## Results

The fragment size of DNA sequencing libraries was set to 500 bp for samples amplified by primer set#1 and from 150 to 1000 bp (median 500 bp) for primer sets 2 and 3. Number of reads obtained for samples amplified by different primer sets differed from 58,103 to 214,804 in average (Table 2).

**Table 2:** Numbers of obtained sequencing reads.

Primers used for library preparation		Number of obtained sequencing reads (data are presented as average of 10 samples ± standard deviation)
set#1	515f/806r	581,030.6 ± 76,301.8
set#2	27f/1492r	171,936 ± 31,566.3
set#3	27f*/1495r	214,809.8 ± 63,342.6

### Microbial diversity evaluation

**Figure 1:**
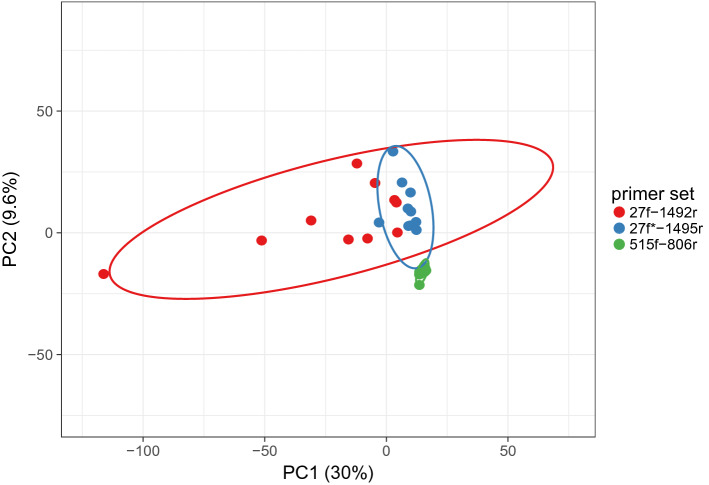
Comparative analysis of bacterial community at genus level of stool samples of children with ASD analysed by three different primer sets visualized by PCA. Analysis was performed at genus level using all detected (2731) bacterial genera. Set#1—515f/806r, set#2—27f/1492r, set#3—27f*/1495r. Unit variance scaling is applied to rows; SVD with imputation is used to calculate principal components. X and Y axis show principal component 1 and principal component 2 that explain 30.4% and 9.7% of the total variance, respectively. Prediction ellipses are such that with probability 0.95, a new observation from the same group will fall inside the ellipse. N = 29 data points.

Totally 2371 different bacterial genera were detected in stool samples of 10 paediatric patients suffering from ASD. With primer set#1—totally 1972 bacterial genera (except of sample C in that 2371 bacterial genera were detected) in average (1181 ± 151), set#2—2223 bacterial genera, in average (1572 ± 120), set#3—2200 bacterial genera, in average (1257 ± 149). Significant difference in number of bacterial genera was detected in comparison of primer set#2/set#1 (p = 6,89095E-05), set#2/set#3 (*p* = 2,63,482-E04), but not set#1/set#3 (*p* = 0.07). For beta diversity determination, PCA analysis of tested samples was carried out. It showed separation into clusters depending on primer sets used for 16S rRNA amplification. The beta diversity determination of analysed samples revealed primer set#2 providing the most divergent data compared to set#1 and set#3. Furthermore, bacterial composition detected with primer set#2 showed the highest variability. On the other hand, group of samples amplified by set#1 was highly “homogenous” (Fig. 1).

### Correlation of used primer set with the bacterial composition of the gut at phylum level

Microbial profiles of samples were analysed at phylum level. In general, microbiota of children with ASD was characterized by high relative abundance of *Bacteroidetes* followed by *Firmicutes* and *Proteobacteria*. In several samples, relatively high relative abundance of *Verrucomicrobia* was detected, however, this was observed only in samples amplified by set#1primers. Except of Bacteria, also low relative abundance (less than 1%) of *Archaea* was detected in samples amplified with set#1 primers (Table 3).

**Table 3:** Relative abundance of bacterial phyla in analysed samples.

Primer sets	Sample	Archea [%]	Actinobacteria [%]	Bacteroidetes [%]	Firmicutes [%]	Proteobacteria [%]	Verumicrobia [%]
515f/806r primers	A	0.3	2	76	19	2	0.04
	B	0.1	9	44	39	5	2
	C	0.1	0.5	61	29	2	7
	D	0.1	0.4	83	15	0.9	0.04
	E	0.1	0.5	63	26	9	0.1
	F	0.1	0.8	65	24	4	6
	G	0.1	0.8	79	18	0.9	0.7
	H	0.8	0.6	73	13	13	0.3
	I	0.1	0.2	59	36	5	0.05
	J	0.1	0.5	89	9	1	0.04
27f/1492r primers	A	0.6	0.4	44	45	9	0.9
	B	0.5	0.6	22	72	3	0.1
	C	0.2	0.6	28	68	2	0.1
	D	0.9	0.4	59	34	5	0.01
	E	0.3	0.5	30	48	20	0.08
	F	1	0.6	38	47	11	0.2
	G	2	0.8	46	39	10	0.3
	H	0.3	0.2	23	17	59	0.03
	I	0.1	0.5	26	47	26	0.04
	J	3	1	30	48	11	0.5
27f*/1495r primers	A	0.06	0.3	58	38	2	0.01
	B	0.07	0.5	25	70	3	0.1
	C	0.07	0.4	36	61	2	0.5
	D	0.1	0.2	66	32	0.9	0.008
	E	0.03	0.2	44	52	3	0.05
	F	0.1	0.2	51	46	2	0.6
	G	0.1	0.3	61	37	1	0.07
	H	0.07	0.2	62	37	0.8	0.02
	I	0.06	0.4	39	59	1	0.02
	J	0.2	0.6	35	60	2	0.04

**Figure 2:**
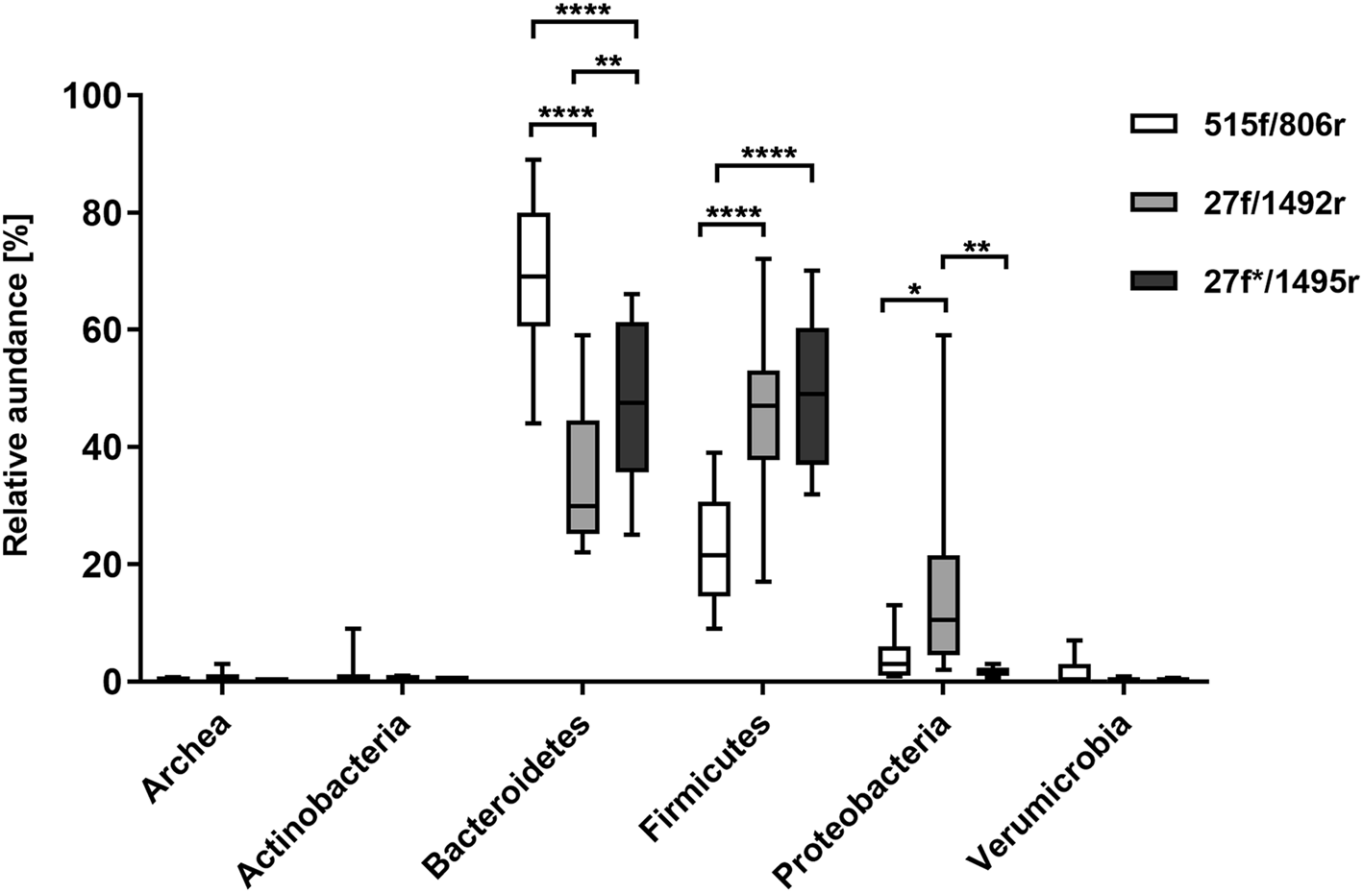
Differences in relative abundance of bacterial phyla in paediatric ASD samples amplified with different primer sets. Primer set#1 (515f/806r) adhered significantly more to *Bacteroidetes* than primer set#2 (27f/1492r) and set#3 (27f*/1495r). For *Firmicutes* detection primer set#3 was the most favourable followed by set#2. Primer set#3 represented balanced ratio of detected OTUs between *Bacteroidetes* and *Firmicutes* phyla. The highest abundance of *Proteobacteria* was detected by primer set#2 compared to set#1 and 3. The level of significance ≤ 0.01 was applied.

Differences in microbial profiles of samples at phylum level were observed depending on set of primers used for DNA sequencing library preparation (Fig. 2). Significantly higher relative abundance of *Bacteroidetes* was detected in microbial profiles of samples amplified by set#1primers, compared to samples amplified by set#2 and set#3 primers (*p* < 0.0001 for both). Difference was observed between samples prepared by set#2 and set#3 primers as well, where samples prepared with set#3 primers had significantly higher *Bacteroidetes* (*p* = 0.0025). On the other hand, in samples amplified by set#2 and set#3 primers, significantly higher abundance of *Firmicutes* was detected (*p* < 0.0001 for both). This observation is in accordance with the ZymoBiomics Gut standard sequencing using primer set#2, where higher amount of *Firmicutes* compared to the original relative abundance defined by the supplier has been determined compared to primer set#1, but not set#3. Significantly higher relative abundance of *Proteobacteria* was detected in samples amplified by set#2 primers compared to samples prepared by set#1 and set#3 primers (*p* = 0.0107 and *p* = 0.0013, respectively).

**Figure 3:**
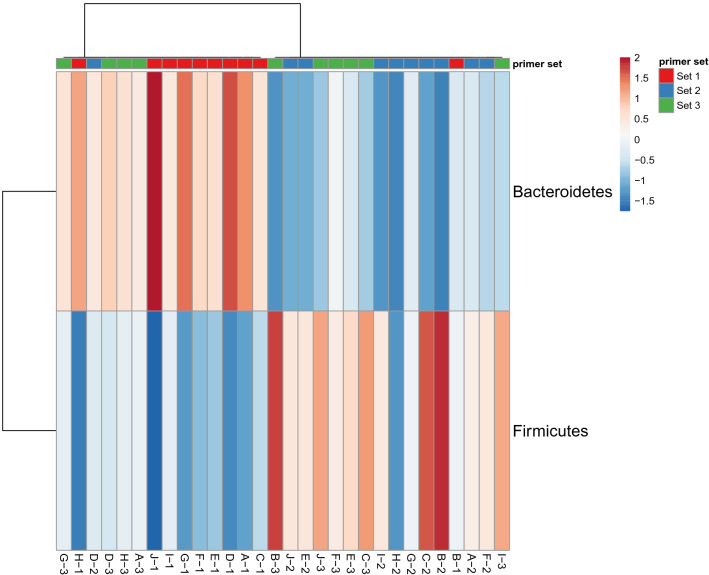
Firmicutes to Bacteroidetes ratio in faecal samples of ASD patients visualized by heatmap. Increased relative abundance of Bacteroidetes compared to Firmicutes in samples in that for amplification of 16S rRNA primer set#1 was used, compared to samples processed with primer set#2 and set#3.

The *Firmicutes* to *Bacteroidetes* ratio (F/B) differed according to primer pair used for analysis. The elevated number of *Bacteroidetes* detected with set#1 mirrored in the lowest F/B ratio 0.37 (0.1–0.9 ± 0.24) that significantly differed from set#2 F/B ratio 1.5 (0.6–3.3 ± 0.9) (*p* = 0.0003) and set#3 F/B ratio 1.2 (0.5–2.8 ± 0.8) (p = 0.001). Between set#2 and set#3 there was no significant difference in F/B ratio (Fig. 3).

### Primer pair effect on detection of microbiome composition at genus level

**Figure 4:**
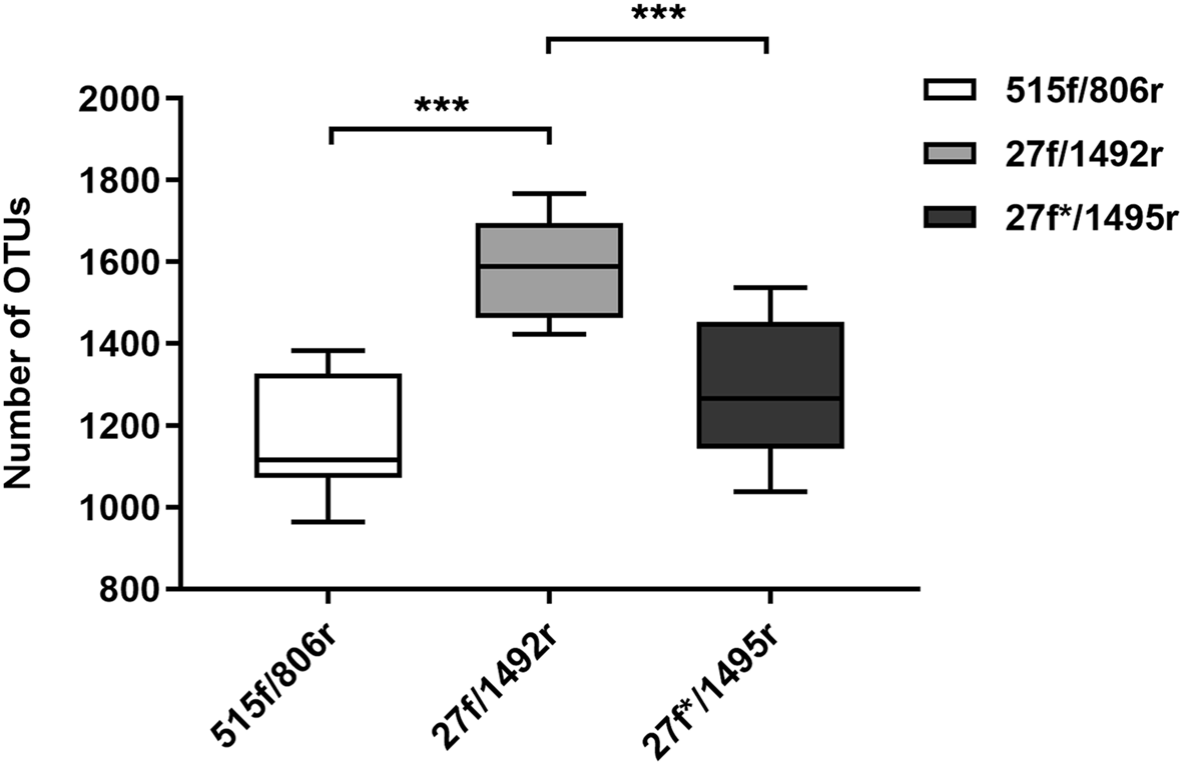
Number of detected bacterial genera in stool samples of paediatric ASD patients determined with three primer sets. Primer set#2 (27f/1492r) matched significantly to more genera than primer set#1 (515f/806r) and set#3 (27f*/1495r). The level of significance ≤ 0.01 was applied.

Major differences were observed also in number of bacterial genera detected in samples amplified by different sets of primers (Fig. 4). The most bacterial genera (1584.7 ± 122.52) were detected in samples amplified by set#2 primers compared to set#1 (1170 ± 145.58 OTUs; *p* = 0.0001) and set#3 primers (1283.6 ± 166.73 OTUs; *p* = 0.0007).

**Figure 5:**
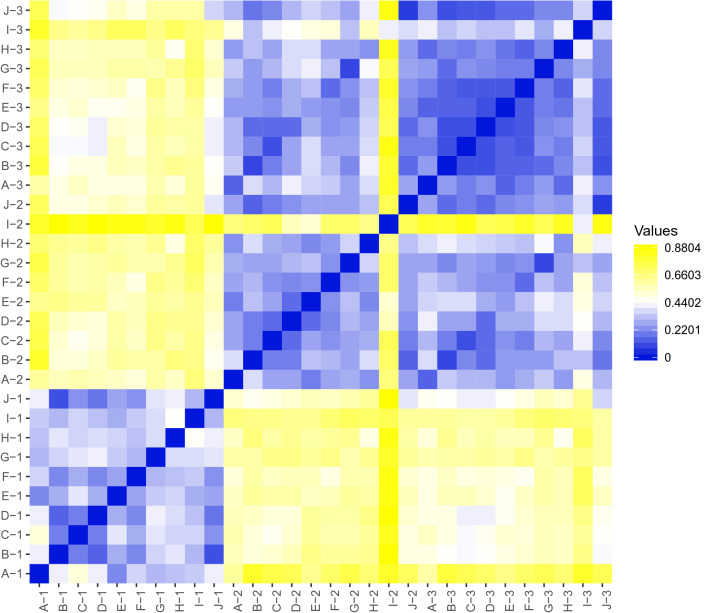
Comparison of relative abundance of bacterial composition between three primer sets amplifying 16S rRNA based on Spearman rank correlation coefficient. The most similar results are obtained with the primer set#2 and primer set#3. For the analysis 244 the most abundant bacterial genera and inclusion criteria for their presence in all samples was applied.

Furthermore, we found out that the microbial composition varied not only between different samples, but it differed also within the same sample amplified with different set of PCR primers. Pairwise analysis and Spearman Rank coefficients of the 244 bacterial genera present in all 10 analysed samples (Fig. 5) showed that the bacterial composition was the most similar between primer set#2 and set#3 (average r of 0. 33) followed by primer set#1 and set#3 (average r of 0.56) and finally set#1 and set#2 (average r of 0.62), regarding the quantity of individual bacterial genera. Within the group of samples amplified with the same primer set, the lowest variability in bacterial taxa abundance was observed with primer set#3 (average of r 0.22) followed by set#1 (average r of 0.32) and set#2 (average r of 0.34).

We observed that detected structure of the microbiota depends directly on primers used for PCR amplification (Table 4). Microbial analysis at genus level showed, that *Bacteroides* was the most abundant genus in 80% of samples. In the remaining 20% of samples, *Prevotella* or *Alloprevotella* dominated. However, we observed differences depending on type of PCR primers used for library preparation. In samples amplified by set#1 primers relative abundance of *Bacteroides* ranged from 1.7 to 63.2% (37.5% ± 19.95%) and *Bacteroides* was the most abundant genus in 7 of 10 samples. We also found out, *Prevotella* to be the most abundant in 2 samples (relative abundance 22.6% and 33.7%) and *Alloprevotella* in 1 sample (relative abundance 42.9%). High relative abundance of *Paraprevotella* 10.9% ± 6.44% was observed in all except one sample prepared with set#1 primers. When set#2 and set#3 primers were used, *Bacteroides* was the most abundant in 9 of 10 samples for both, and its relative abundance was 22.7% ± 10.93% and 32.6 ± 16.21%, respectively. *Faecalibacterium* was highly abundant in all samples and the relative abundance only slightly differed depending on primer set used for library preparation. In most of samples prepared with primer set#2 and 3 *Clostridium* was highly abundant, however, only in 1 sample prepared by set#1 primers was more represented. High abundance of *Salmonella* and *Escherichia/Shigella* was found in 3 and 2 samples correspondingly, when using set#2 primers, but this was not observed in the same samples prepared by different sets of primers (Table 4).

**Table 4:** The list of the most abundant bacterial genera in samples analysed using different type of 16S rRNA primer sets.

Top 10 genera	515f/806r PCR primers		27f/1492r PCR primers		27f*/1495r PCR primers	
	Number of samples (n = 10)*	Relative abundance [%]**	Number of samples (n = 10)*	Relative abundance [%]**	Number of samples (n = 10)*	Relative abundance [%]**
*Bacteroides*	10	35.68 ± 18.92	10	22.69 ± 12.16	10	32.62 ± 15.38
*Faecalibacterium*	10	3.73 ± 2.14	10	4.8 ± 3.07	10	5.47 ± 2.93
*Roseburia*	5	1.5 ± 0.44	6	5.1 ± 0.92	7	4.59 ± 2.02
*Paraprevotella*	9	10.91 ± 6.07	2	2.48 ± 0.86	3	3.54 ± 1.04
*Alistipes*	6	2.53 ± 1.29	3	2.16 ± 0.39	4	2.5 ± 0.63
*Dialister*	3	2.75 ± 2.1	4	7.2 ± 4.36	5	6.77 ± 4.86
*Clostridium XlVa*	0	–	5	1.68 ± 0.31	6	1.87 ± 0.43
*Lachnospiracea*	0	–	5	2.4 ± 0.68	6	2.14 ± 0.64
*Prevotella*	3	33.09 ± 8.3	4	12.51 ± 6.86	4	16.69 ± 9.33
*Blautia*	4	1.75 ± 1.08	2	2.08 ± 0.32	4	1.83 ± 0.31
*Parabacteroides*	3	3.91 ± 1.19	3	3.31 ± 1.47	4	4.43 ± 2.32
*Phascolarctobacterium*	3	3.61 ± 0.22	3	7.32 ± 4.24	3	5.81 ± 2.85
*Veillonella*	3	4.55 ± 5.82	3	4.16 ± 4.19	2	6.66 ± 5.36
*Clostridium IV*	1	4.07 ± 0	3	4.13 ± 0.82	3	4.3 ± 1.17
*Ruminococcus*	1	2.62 ± 0	3	5.58 ± 1.46	3	5.53 ± 2.21
*Gemmiger*	0	–	2	2.06 ± 0.33	4	1.47 ± 0.27
*Oscillibacter*	0	–	3	2.34 ± 0.75	3	2.28 ± 0.75
*Alloprevotella*	3	25.16 ± 12.6	1	2.31 ± 0	1	3.02 ± 0
*Orenia*	5	2.28 ± 1.68	0	–	0	–
*Acidaminococcus*	2	2.71 ± 1.57	1	5.95 ± 0	1	6.18 ± 0
*Barnesiella*	2	2.42 ± 0.21	0	–	2	1.52 ± 0.15
*Eisenbergiella*	0	–	2	2.14 ± 0.55	2	2.7 ± 0.34
*Anaerofilum*	0	–	1	2 ± 0	2	1.55 ± 0.13
*Anaerostipes*	0	–	1	1.75 ± 0	2	2.35 ± 0.55
*Pantoea*	0	–	3	3.48 ± 2.45	0	–
*Salmonella*	0	–	3	5.66 ± 3.91	0	–
*Akkermansia*	2	6.36 ± 0.44	0	–	0	–
*Desulfonispora*	2	3.33 ± 0.09	0	–	0	–
*Escherichia/Shigella*	0	–	2	9.81 ± 5.72	0	–
*Oribacterium*	2	2.39 ± 0.17	0	–	0	–
*Yersinia*	0	–	2	2.81 ± 1.15	0	–

*The number of samples in which bacteria belonged to the 10 most abundant.

**Relative abundance of bacteria in total microbiota of samples in which bacteria belonged to the 10 most abundant (Data are presented as average ± standard deviation).

### Analysis of the least abundant bacterial genera

**Figure 6:**
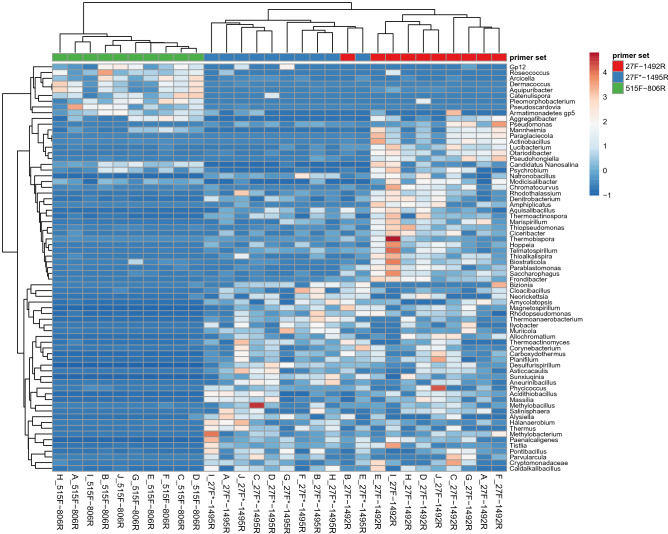
Comparison of the detection capability of three primer sets based on the least abundant bacterial genera with significantly different abundance among groups. For data analysis student´s t-test was performed. Bacterial genera with significantly altered abundance between compared datasets (*p* ≤ 0.005) were visualized using heatmap.

Of the whole number of bacteria detected by individual primer sets (app.1500 genera per sample), the least abundant 500 genera of each primer set group were selected. Next, only genera significantly different (p < 0,005) between the sets of taxa obtained with different primer sets (71 genera) were considered for further analysis. Comparison of these groups revealed that primer set#2 (27f/1492r) is able to detect the most of unique bacterial genera representing less than 0.0001% of the detected bacterial community (in average 62 of 71) (Fig. 6). Furthermore, it detected the same group of bacterial genera like the primer set#3. This set of bacteria is represented also by *Aggregatibacter, Pseudomonas, Mannheimia, Paraglaciecola, Actinobacillus, Lucibacterium, Otariodibacter, Pseudohongiella, Candidatus Nanosalina, Psychrobium, Natronobacillus, Modicisalibacter, Chromatocurvus, Rhodothalassium, Denitrobacterium, Amphiplicatus, Aquisalibacillus, Thermoactinospora, Marispirillum, Thiopseudomonas, Ciceribacter, Thermobispora, Hoppeia, Telmatospirillum, Thioalkalispira, Biostraticola, Parablastomonas, Saccharophagus* and *Frondibacter*. On the contrary, primer set#1 detected in average 13 unique bacterial genera including *Gp12, Roseococcus, Arcicella, Dermacoccus, Aquipuribacter, Catenulispora, Pleomorphobacterium, Pseudoscardovia, Armatimonadetes gp5, Pseudomonas, Candidatus Nanosalina, Psychrobium* and *Modicisalibacter* differing from bacteria detected with set#2 or set#3.

### Detection of ASD-associated bacterial genera

**Figure 7:**
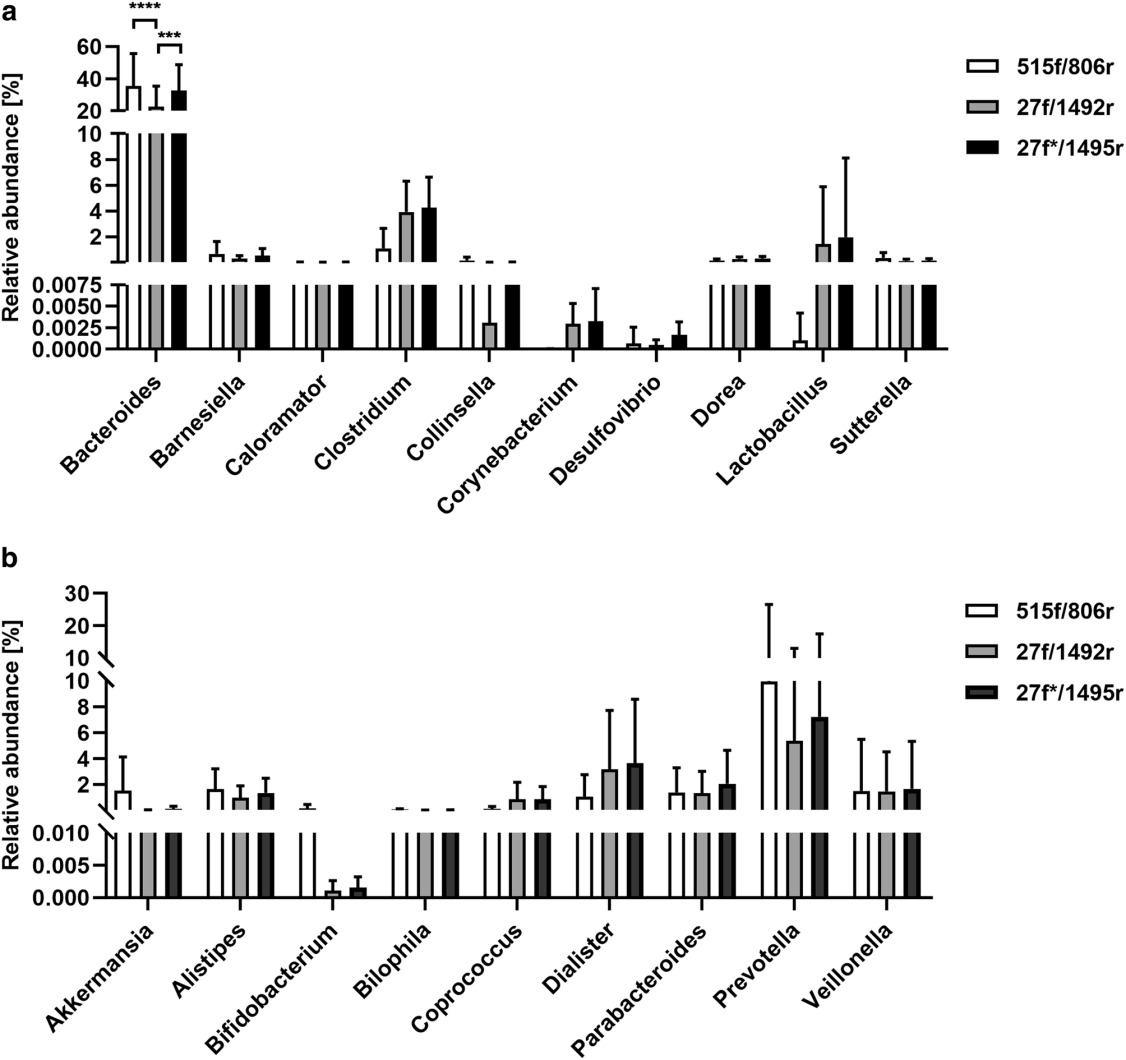
(**a**) Relative abundance of bacterial species that have been observed to be elevated in children with ASD. The level of significance ≤ 0.01 was applied. (**b)** Relative abundance of bacterial species that have been observed to be decreased in children with ASD. The level of significance ≤ 0.01 was applied.

Several bacterial species have been observed in previous studies to have elevated or decreased abundance when compared samples of children with ASD and neurotypical controls^27,34,35,43,47–55^. We analysed ability of 3 different primer sets to detect bacterial genera often associated with ASD. We observed significantly higher ability to detect *Bacteroides* by set#1 and set#3 rather than set#2 (*p* < 0.001 and *p* = 0.002 respectively). There was also a trend for higher abundance of *Clostridium*, *Corynebacterium*, *Lactobacillus*, *Coprococcus* and *Dialister* in set#2 and set#3 prepared samples. In contrast, samples amplified by set#1 showed higher relative abundance of *Akkermansia*, *Bifidobacterium* and *Prevotella*. For the detection of *Colinsella* and *Alistipes* rather primer set#1 and set#3 seem to be more suitable than set#2 (Fig. 7a,b).

## Discussion

Intestinal microbiota analysis of an individual may be very variable since it is influenced by many factors. For example, false differences may be introduced during sample processing, different results may be obtained when different protocols for DNA library preparation or data analysis are used. Preparation procedures as well as sequencing procedures might be very important issue when comparing results of different experiments. Therefore, unification of procedures in studying of microbiota composition plays an important role.

In our study, clear differences were observed depending on type of DNA sequencing library preparation method. In samples amplified with set#1 primers, *Bacteroidetes* were definitely the most abundant in microbiota of children with autism. This is in line with previously published study of Finegold et al.^55^ who observed *Bacteroidetes* to be the most abundant in autistic microbiota, followed by *Firmicutes*, similarly to our study. In our primer pair evaluation the closest result was obtained by amplification of the shortest PCR product, only the V4 region of *16S rRNA* gene compared to primer set used in Finegold´s analysis (amplification of V4-V6 region)^55^. The primer sets 2 and 3 gave rather opposite Bacteroidetes:Firmicutes ratio or even more abundant *Firmicutes* using the shot-gun sequencing of total *16S rRNA* gene. The used ZymoBiomics Gut Microbiome Standard enabled us to validate not only the library preparation protocol, but also the DNA isolation method as well as to filter out wet-lab and dry-lab contamination from samples. In summary, shot-gun sequencing of pcr amplicon encompassed all the bacteria included within the recommended standard including *Firmicutes*, *Bacteroidetes*, *Verrucomicrobia*, *Fusobacteria* and *Proteobacteria*. The only genus *Bifidobacterium* (*Actinobacteria*) was detected by V1-V9 sequencing approach with 100-times lower abundance compared to the original mock community. Here we could speculate that it is a matter of GC content (59.2%) of *Bifidobacterium adolescentis*. However, the *Firmicutes* represented by bacterial species with wide range of GC content (*Faecalibacterium* 57.8%; *Veillonella* 39.0%; *Roseburia* 48.7%; *Lactobacillus* 52.3%; *Clostridioides* 28.8%) were identified almost equally to original ratio within mock community and detected without any preference for the GC content. So, we suppose that the GC content of the *16S rRNA* gene could be more balanced than of the whole genome, at least for Firmicutes. What can rather be influenced is the preference of primer set#2 and set#3 for *Firmicutes* than for *Bacteroidetes*, since the GC content of the represented bacteria (*Bacteroides* 43.3%; *Prevotella* 44.4%) was higher than of some of the *Firmicutes* representatives or similar. The ratio of *Firmicutes* to *Bacteroidetes* was favourable for *Firmicutes* what matched the premixed standard (50% *Firmicutes*, 26% *Bacteroidetes*) even though the relative abundance of *Bacteroidetes* was lower than expected (5-times). This is an important point for data validation using qPCR methods, since it is based on short amplicons amplification, and as such opposite results can be obtained compared to 16S sequencing based on full-length *16S rRNA* sequence. Real-time PCR analysis belongs still to the most widely used methods for bacterial analysis also in terms of their quantification^27,56^ so careful primer set selection needs to be taken into account according to further intents.

At genus level, *Bacteroides* and *Faecalibacterium* were present across all samples with high relative abundance. This is in line with previously published studies^34,55^. Except these genera, in Finegold study, *Clostridium*, *Eubacterium*, *Ruminococcus*, *Roseburia*, *Akkermansia*, *Parabacteroides* and *Alistipes*, were also the most abundant^55^. In another study, where bacterial 16S rRNA genes were amplified using a primer set specific for V3–V5, genus level analysis showed different top ten most abundant genera *Bifidobacterium*, Unknown *Lachnospiraceae*, *Blautia*, *Ruminococcus*, *Clostridium XI*, *Streptococcus*, *Gemmiger*, and *Lachnospiraceae incertae sedis*^34^. On contrary with previously published study, we did not confirm high abundance of *Bifidobacteria* and *Eubacterium*. For other bacteria, we observed that relative abundance was depending on type of DNA library preparation and varied among samples. However, none of above mentioned species were highly abundant in more than half samples in our study, except *Alistipes* which was among 10 most abundant species in 6 samples prepared by set#1primers, *Roseburia* highly present in 7 samples prepared by 27f*/1495r primers and *Lachnospiracea* with high relative abundance in 6 samples prepared by 3 set of PCR primers.

Higher levels of *Clostridium*, *Suterrella* and *Ruminococcus* are considered to be associated with pathogenesis of ASD. Some previously published studies, confirmed increased abundance of *Clostridia* compared to healthy controls^53,54^ as well as increased *Suterrella* and *Ruminococcus*^51^. In our study, we observed differences in relative abundance of *Clostridium* depending on type of sequencing library preparation. *Clostridium* was detected with high abundance only in 1 sample amplified by 1 set of primers. However, *Clostridia* were present in high relative abundance in samples amplified by second and third set of PCR primers. On the other hand, *Suterella* was not found to be among the most abundant species in any sample and *Ruminoccocus* was detected to be among 10 most abundant species in half of samples amplified by 2 and 3 set of primers, and only in 1 sample amplified by 1 set of primers.

Previously published studies confirmed that many factors may influence results of sequencing analysis. These include storage conditions, DNA extraction method, type of primers used for DNA amplification and sequencing, sequencing platform and chemistry, as well as data analysis, which have an impact on detected microbiota composition^57–60^. The studies also indicate, that only suitable combination of several factors may result in findings that truly reflects reality. Although several primer sets are commonly used and considered to be universal, different results may be obtained. Study comparing V4, V6-V8 and V7-V8^57^, as well as other study comparing V4-V5, V1-V2 and V1-V2 degenerate primers^58^, both confirmed, that V4/V4-V5 primers give results that are the most comparable across platforms and the least biased. On the other hand, V4-V5 regions resulted in lowest number of analysable reads compared to others. Using V4 primers (515f/806r primers) in our study resulted significantly higher *Bacteroides* and lower *Firmicutes* than by two others primer sets, as well as significantly lower abundance of Proteobacteria than detected by 27f/1492r amplification primers.

In study of Farris and Olson universal primer sets (24F and 1492R original and modified versions of primers specific for *Actinobacteria*) for 16S rRNA genes amplification were used. These primers were not able to amplify 20%-50% of isolates. In this study, 1492R provided more effective identification of *Actinobacteria* than 1492-modif^61^. In our study, we did not observe significant differences in ability of 3 primer sets to identify *Actinobacteria* in samples. On the other hand, it is clear, that in our experiment, *Actinobacteria* presented only low percentage of microbial communities. This might be supported by fact, that some bacteria especially those whose sequences include high G + C content (also characteristic for *Actinobacteria*) are usually identified in lower abundance than in real microbial communities^62^.

Though only minor changes in sequences of 27f*/1495r primer set compared to standard set of 27f/1492r primers, in several phyla significant differences in microbiota composition were detected. We found out significantly higher relative abundance of *Bacteroidetes* and significantly lower relative abundance of *Proteobacteria* when samples were amplified by modified (27f*/1495r primers) compared to samples amplified by standard primers (27f/1492r primers). Interestingly, when it comes to microbial composition on genus level, only minor differences were observed between samples amplified by set#2 and set#3 of primers.

## Conclusion

We have found out, that the primer set 27f/1492r (primer set#2) can provide much wider information regarding the diversity of samples from ASD children and can provide deeper insight into intra-individual variability. On the other side, the 16S rRNA V4 region can provide very homogenous information on bacterial composition typical for ASD patients what in comparative studies is an invaluable tool for discriminative analysis. Here we prove also that the selection of 16S rRNA variable regions for analysis matters more than the primer set itself in terms of bacterial semiquantitative analysis, based on the comparative analysis of primers 27f*/1495r (set#3) and primers 27f/1492 (set#2). However, the most interesting is the finding, that the *Firmicutes/Bacteroidetes* ratio highly depends on the analysed 16S rRNA region; the 27f/1492r provides approximately 4-times higher F/B ratio than 515f/806r what points to the bottle-neck of this value in any microbiome analysis.
